# High prevalence of vitamin D deficiency among the South Asian adults: a systematic review and meta-analysis

**DOI:** 10.1186/s12889-021-11888-1

**Published:** 2021-10-09

**Authors:** Mahbubul H. Siddiqee, Badhan Bhattacharjee, Umme Ruman Siddiqi, Mohammad MeshbahurRahman

**Affiliations:** 1 School of Data and Sciences, BRAC University, Dhaka, 1212 Bangladesh; 2 Research and Development Wing, Red and White Innovations, Dhaka, 1216 Bangladesh; 3 Communicable Disease Control Unit, Directorate General of Health Services, Dhaka, 1212 Bangladesh; 4 Biomedical Research Foundation, Dhaka, 1230 Bangladesh

**Keywords:** Vitamin D deficiency, South Asia, Adults, Systematic review

## Abstract

**Background:**

Vitamin-D deficiency is linked to a wide range of chronic and infectious diseases. Body of literature suggested that the prevalence of this deficiency can have geographical variation. Although vitamin D deficiency is frequently reported in the South Asian population, the scarcity of systematic reviews and meta-analysis means the true extent of the disease and the underlying factors causing it are poorly characterized.

**Methods:**

A systematic search was performed using two databases (PubMed and Scopus) and one search engine (Google Scholar) for original studies on the South Asian population (published from January 1, 2001, to December 31, 2019). Following the search, a random effect meta-analysis was performed to calculate population-level weighted average, the pooled prevalence of deficiency, and heterogeneity of vitamin D among different countries and genders; in addition to South Asia as a whole.

**Results:**

Our study, based on our selection criteria was narrowed down to a total of 44,717 participants; which spanned over 65 studies from five South Asian countries. Overall, the pooled prevalence of deficiency was 68% [95% CI: 64 to 72%] with significant heterogeneity (I^2^ = 98%; *p* = 0.00). The average level of vitamin D ranged from 4.7 to 32 ng/mL, with a weighted mean of 19.15 ng/mL (weighted standard deviation 11.59 ng/mL). The highest prevalence of vitamin D deficiency was found in Pakistan (73%;95% CI: 63 to 83%) followed by Bangladesh (67%; 95% CI: 50 to 83%), India (67%; 95% CI: 61 to 73%), Nepal (57%; 95% CI: 53 to 60%) and Sri Lanka (48%; 95% CI: 41 to 55%), respectively. This finding indicated a high degree of heterogeneity among the population. (I^2^ = 98.76%), Furthermore, a gender-wise analysis suggested that in South Asia, the prevalence of vitamin D deficiency was higher in females than males.

**Conclusion:**

Our findings reveal highly prevalent and variable vitamin D deficiency among the adults of different South Asian countries. Findings from this review would be helpful to generate hypotheses and explore the factors affecting the inter-country variability, alongside strengthening evidence for governments to prioritize mitigation strategies in this region.

**Supplementary Information:**

The online version contains supplementary material available at 10.1186/s12889-021-11888-1.

## Background

The critical roles of vitamin D in several endocrine, paracrine, and autocrine activities have been specified in various studies [1–3]. Vitamin D deficiency in adults acts as a key factor for the development of various communicable and non-communicable diseases. For instance, inadequate levels of vitamin D play a major role for the development of diseases like colon cancer, breast cancer, cardiovascular diseases, diabetes mellitus, multiple sclerosis, rheumatoid arthritis, Parkinson’s disease, tuberculosis etc. [1–4]. Despite the high physiological importance, deficiency of this vitamin is commonly reported around the world.

Study reports suggest that approximately 1 billion people worldwide are affected with vitamin-D deficiency and around 50% of the global population have vitamin D insufficiency [1, 3]. According to literature, the prevalence of vitamin D deficiency in Europe, USA and Middle East has been reported to range from 20 to 90% [5, 6]. Similar trends have been reported in countries like Australia, India, Africa, South America, Turkey and Lebanon [1, 3, 5, 6]. These reports suggest that insufficient vitamin D is a problem for both developing and developed countries.

Alongside high prevalence, a wide variation of vitamin D status exists among different countries. For example, study reports showed that adult population in the Middle Eastern countries such as Iran and Syria have very low average level of vitamin D (14 ng/mL and 10 ng/mL, respectively) [7, 8], compared to the adults in the European countries like Denmark and France (26 ng/mL and 24 ng/mL, respectively) [9, 10].

A number of factors have been highlighted as underlying variables to explain this world-wide variation of vitamin D status among different populations. Degree of sunlight exposure and the factors that influence its duration and intensity ultimately influence the level of vitamin D in any population [1–3]. Among such factors, geographical location is key [11]. However, alongside sunlight exposure, skin color, age, comorbidities are also reported as modulating factors to explain variations of vitamin D levels, among populations [1–3, 11]. Therefore, understanding the comparative variability and diversity of prevailing factors that may influence levels of vitamin D among populations, is imperative from a public health standpoint.

South Asia (SA) occupies eight nations and these nations are Bangladesh, India, Pakistan, Nepal, Bhutan, Maldives, Sri Lanka and Afghanistan. These eight nations have a total population of around 1.8 billion, representing over 24% of the total world population [12, 13]. Although, the individual SA countries share some common ethnic and cultural characteristics, differences are observed for language, religion clothing practice, food habit etc. [14, 15].

Communicable and non-communicable diseases, nearly all of which are associated with vitamin D deficiency, are highly prevalent among the SA population [16–18]. Despite the possibility of vitamin D being a potential risk factor, we found only one systematic review on the adult population (Indian population only yet on quantitative measurement of serum vitamin D concentration, not percentage prevalence of deficiency) [19]. Thus, a huge knowledge gap exists about the true extent of vitamin D deficiency in different SA countries. This systematic review intends to address this knowledge gap through the performance of a meta-analysis.

## Methods

We followed the preferred method of reporting items for systematic reviews and meta-analyses (PRISMA-P 15) guidelines, for conducting this systematic review [20].

### Data source and search strategy

Two main databases (PubMed and Scopus) and one search engines (Google Scholar) were systematically explored for original articles on vitamin D deficiency of SA adult population (After singing out of all Google accounts, a search was performed to avoid personalized results). The search was done independently by two researchers (BB and MMR) (from October 12, 2019 to January 12, 2020) to find out the studies conducted from 2001 until the search date. The details of the search strategy, list of original MeSH terms and the alternative terms (to minimize chance of exclusion) used in this study is provided in the additional file (Table- A1).

To maximize the search efficiency, corresponding authors profiles (on Google Scholar, Research Gate, Orchid, and in current organizations) and reference list of our selected studies were also explored. To ensure the inclusion of grey literature in this review, we went through online archives of newspapers which are published in English language among SA countries such as Times of India, The Daily star, Dawn, Daily Bhutan, Maldives insider, The Himalayan times, Daily Mirror etc. Government reports and published abstracts (in electronic version) from conferences held in the SA countries were also explored as relevant sources.

### Eligibility criteria

In this study, vitamin D deficiency was defined as per the definition proposed by the Clinical Practice Guideline of the Endocrine Society (that recommends < 20 ng/mL as cut off) [21] Studies were selected if they reported vitamin D deficiency of SA adults. The inclusion criteria were- 1) study subjects aged 18 years or above; 2) original peer reviewed observational studies (cross-sectional, longitudinal, case-control (control groups only), randomized clinical trial (baseline/placebo groups only); 3) study conducted on community level or in hospital settings on apparently healthy population (people with minor illness whose physical condition were not correlated with any chronic disease); 4) study conducted in SA countries (Bangladesh, India, Pakistan, Nepal, Bhutan, Maldives, Sri Lanka and Afghanistan); 5) and, study conducted from January 1st, 2001 to December 31st, 2019.

The following exclusion criteria were applied- 1) studies (or groups) that had sample size less than 50 [22]; 2) reported vitamin D levels after some form of intervention or supplementation; 3) conducted on groups other than healthy adults (pregnant women, infants, neonates and adolescents); 4) reported prevalence of vitamin D deficiency associated to any kind of disease (chronic kidney, liver and heart disease, cancer, diarrhea, anemia, diabetes) or disease related to any coexisting morbidity (body aches and pain, proximal muscle weakness, osteoporosis); 5) conducted on special group of population such as short stature or mentally retarded; 6) did not report prevalence of vitamin D deficiency and mean level of vitamin D status; 7) studies which met selection criteria but the full text could not be retrieved from the authors after requests.

Mendeley Desktop software (version 1.19.4) was used to manage the references and duplications. After checking duplications, papers were independently verified by two researchers (MHS and URS) before final inclusion in meta-analysis. Disagreements were resolved through discussion with co-authors.

### Data extraction

A standardized data table was used to extract data from all eligible studies. For each study, the following information was extracted: publication details [e.g., first author, publication date, journal name, and publisher]; research setting (community-based or hospital-based); population and study design [e.g., country, study area and sample size]; participants’ characteristics and major findings [e.g., gender, socio-economic status, method of measurements, mean level of vitamin D and prevalence percentage of deficiency]. All investigators had checked the accuracy of the data extracted through multiple revisits to the included articles.

In our selected studies, where mean values of 25(OH) D were given in nanomol per unit liter (nmol/L), we converted to nanogram per milliliter (ng/mL) by dividing with 2.5 (according to the international unit conversion system) to maintain the uniformity. Since the study’s outcome of interest included the prevalence of vitamin D deficiency in SA countries, vitamin D prevalence and mean value was only extracted after reading the selected papers in full.

### Evaluation of study quality

The risk of bias for each selected study were assessed by Badhan using Hoy et al. [23] guidelines on conducting prevalence and incidence reviews. If the studies full-fill any criteria with a score of 0 it implies a low risk of bias, and if studies full fill a criteria with score of 1 this indicates a high risk of bias. Risk of bias was assessed for all original articles.

### Statistical analysis

Mean value of participant’s vitamin D status and prevalence of vitamin D deficiency in SA countries were considered as summary measurements. According to literature, serum level of vitamin D may depend on a number of confounding variables (e.g., geolocation, gender, age, socioeconomic status, clothing practices, skin complexion, etc.) and their effects are likely to vary among different populations [1–3, 11]. Since the individual studies included in our final data set did not report the effects of all these variables, we used random-effect model to obtain pooled prevalence with a 95% confidence interval [24–26]. Country-wise and gender-wise analysis was also performed according to the vitamin D deficient participants. Heterogeneity was assessed using Cochran’s Q test and the I^2^ statistics. Substantial heterogeneity was indicated with an I^2^ of more than 75% [27]. Publication bias and small study effects were also examined using funnel plot and Egger’s test. All statistical analyses were conducted by Stata version 15 (Stata Corp, College Station, TX) using the ‘metaprop’, ‘metabias’, ‘metafunnel’ commands. Weighted mean level of serum vitamin D was calculated in Microsoft Excel (version; 2016).

## Results

**Fig. 1:**
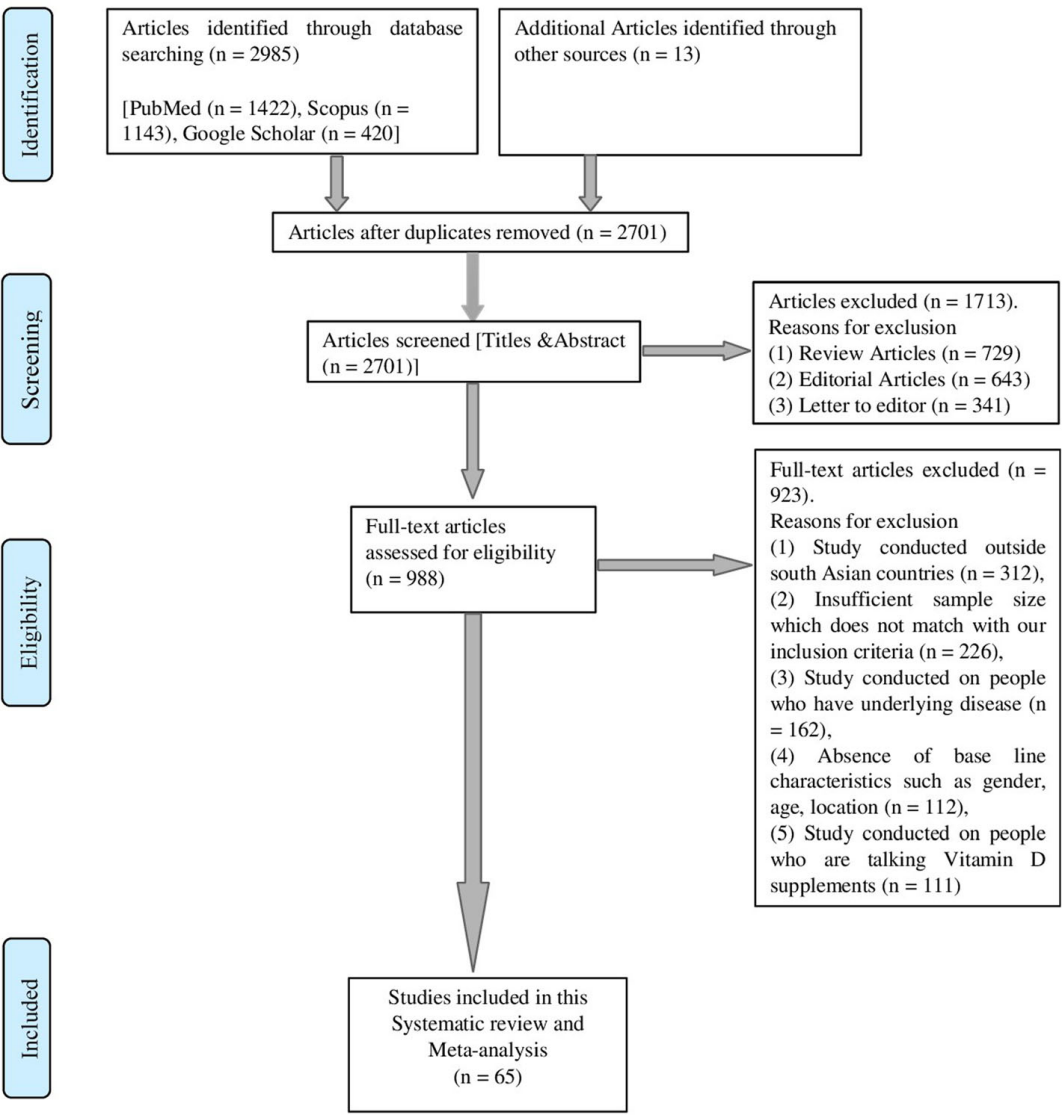
PRISMA chart showing the summary of search result and selection of studies for final analysis

Our systematic search retrieved 2998 study articles from different databases by using the search strategy elaborated earlier. Among these 2998 articles, 2933 were excluded because of being unable to fully fill our inclusion criteria. Finally, 65 study articles were selected. Study article selection process is shown in (Fig. 1).

Out of 65 studies, 26 studies did not mention the demographic area for the study of the population. The remaining studies included it, where 3 studies were conducted on the rural population, 25 studies on the urban population, and 11 studies were conducted on both rural and urban population. More than half of the studies were hospital based (35 out of 65) and rest of them were community based. Socio economic status for the study population was not mentioned in majority of the studies (42 out of 65). For socio economic groups of the population, 5 studies were conducted on lower economic groups and a single study was conducted on the upper and middle-income groups of the population. The rest of them were conducted on a mixed population which represents the lower, middle and upper economic groups of the population. Maximum number of study designs were cross sectional (55 out of 65) and the rest of them were either case control or randomized control trials. Several kinds of measurement methods were used to determine vitamin D status like ELISA (Enzyme linked immunosorbent assay) RIA (Radioimmunoassay), Chemiluminescent Immunoassay (CLIA), Chemiluminescence micro particle Immunoassay (CLMA), HPLC (High performance liquid chromatography), Electro chemiluminescent immunoassay etc. Among these RIA and ELISA were mostly used (33out of 65). Only four studies did not mention the method of vitamin D estimation [28–31]. A Summary outlining the characteristics of selected articles is presented in (Table 1).

**Table 1:** Characteristics of selected study articles

Authors	Year	Country	Study area (urban or rural)	Study design	Age	Gender	Socio economic status	Vitamin D estimation method	Sample size (N)	Average level of vitamin D (ng /mL)	Standard deviation (S.D)
Arya et al. [32]	2003	India	Urban	Hospital – CS	24–53 y	Both	NM	RIA	92	12.3	10.9
Malhotra et al. [33]	2009	India	NM	Hospital – RCT	18 y or above	Female	NM	RIA	100	4.7	3.4
Harinarayan et al. [34]	2011	India	Urban	Hospital – CS	Mean age 38 y	Female	NM	RIA	55	15.7	10.23
Agrawal et al. [35]	2013	India	NM	Hospital – CS	50 y or above	Male	NM	RIA	200	18.96	10.23
Vupputuri et al. [36]	2006	India	Urban	Community – CS	Mean age 43.3 y	Both	Middle	RIA	105	9.8	6
Suryanarayanaet al [37]	2018	India	Urban	Community – CS	60 y or above	Both	Both	RIA	298	19.3	9.32
Shivane et al. [38]	2011	India	Urban	Community – CS	25–35 y	Both	NM	RIA	1137	17.4	9.1
Harinarayan et al. [39]	2006	India	Both	Community – CS	Mean age 44.5 y	Both	Both	RIA	1146	21.135	0.8
Shukla et al. [40]	2016	India	NM	Hospital – CS	More than 20 y	Both	NM	ECLIA	26,273	21.44	14.06
Sanket et al. [41]	2016	India	NM	Hospital – CC	18–65 y	Both	NM	CLIA	81	26.25	NM
Karoli et al. [42]	2014	India	Both	Hospital – CS	18–60 y	Both	Lower	ECLIA	100	30.6	10.8
Laway et al. [43]	2014	India	NM	Hospital – CC	More than 25 y	Both	NM	RIA	102	28.46	18.89
Gupta et al. [44]	2014	India	NM	Hospital – CC	35 y or more	Both	NM	CLIA	70	26.32	14.12
Bhatt et al. [45]	2014	India	Urban	Community – CS	18–60 y	Both	Both	RIA	137	18.9	6.7
Marwaha et al. [46]	2011	India	Urban	Community – CS	More than 50 y	Both	NM	RIA	1346	9.85	7.73
Goswami et al. [47]	2009	India	NM	Community – CS	15–60 y	Both	NM	RIA	642	7	4.08
Zargar et al. [48]	2007	India	Both	Community – CS	18–40 y	Both	NM	RIA	92	10.286	8.215
Harinarayan et al. [49]	2004	India	Both	Community – CS	Mean age 45 y	Both	NM	RIA	316	16.76	5.61
Sofi et al. [50]	2017	India	Both	Hospital – CS	20–49 y	Female	Both	CLIA	224	10.55	7.65
Beloyartseva et al. [51]	2012	India	Urban	Community – CS	Mean age 42.7y	Both	NM	RIA	2119	14.35	10.62
Singh et al. [52]	2018	India	Both	Hospital – CS	20–50 y	Female	NM	CLIA	72	22.91	16.18
Shetty et al. [53]	2014	India	Urban	Community – CS	50 y or above	Male	NM	CLIA	252	20.4	8.3
Kajale et al. [54]	2015	India	Urban	Hospital – CS	Mean age 27.7 y	Female	Both	ELISA	300	15.76	8.8
Goswami et al. [55]	2008	India	Rural	Community – CS	Mean age 42.8 y	Both	NM	RIA	57	14.56	9
Kiran et al. [56]	2014	India	Rural	Community – CS	20–72 y	Both	Both	CMIA	81	15.49	7.58
Kumar et al. [57]	2017	India	NM	Hospital – CC	Mean age 48.2y	Both	NM	CLIA	150	30.6	10.2
Goswami et al. [58]	2016	India	Urban	Community – CS	Mean age 25–32 y	Male	NM	CLIA	194	19.66	6.17
Meena et al. [59]	2016	India	Urban	Hospital – CS	NM	Female	Lower	RIA	100	6.3	4.6
Singh et al. [60]	2011	India	NM	Hospital – CS	Mean age 28 y	Male	NM	RIA	80	9.1	6.54
Garg et al. [61]	2013	India	Urban	Community - CS	50 y or above	Both	NM	RIA	1346	9.8	7.6
Harinarayan et al. [62]	2011	India	Urban	Hospital – CS	Mean age 53 y	Female	NM	RIA	136	17.7	11.08
Paul et al. [63]	2008	India	Urban	Community - CS	50 y or above	Female	NM	RIA	150	20.85	8.63
Srimani et al. [64]	2017	India	Rural	Community - CS	45–70 y	Female	NM	EIA	222	20	NM
Harinarayan et al. [65]	2004	India	NM	Community - CS	Mean age 54 y	Female	NM	RIA	164	14.6	7
Agarwal et al. [66]	2013	India	Urban	Hospital - RCT	40–73 y	Female	High	RIA	92	13.43	8.67
Mitra et al. [67]	2016	India	NM	Hospital – CS	45–52 y	Female	NM	CLIA	64	19.5	18.92
Agarwal et al. [68]	2014	India	NM	Hospital – CS	Mean age 56.3 y	Female	NM	CK	71	12.73	7.63
Dixit et al. [69]	2018	India	NM	Hospital – CS	Mean age 56.4 y	Female	NM	RIA	334	12.95	8.08
Kadam et al. [70]	2010	India	Urban	Hospital – CS	40–75 y	Female	Both	RIA	172	10.22	5.68
Mahmood et al. [71]	2017	Bangladesh	Both	Community - CC	20–40 y	Female	Lower	CMIA	80	18.3	2.5
Islam et al. [72]	2007	Bangladesh	Urban	Community - CS	18–36 y	Female	Lower	EIA	200	14.68	4.48
Islam et al. [73]	2006	Bangladesh	Urban	Community - CS	18–60 y	Female	NM	RIA	66	12.3	4.52
Islam et al. [74]	2002	Bangladesh	Both	Community - CS	16–40 y	Female	Both	RIA	189	16.04	NM
Acherjya et al. [75]	2019	Bangladesh	Both	Hospital – CS	10–70 y	Both	NM	RAIA	160	18.6	6.59
Mubashir et al. [76]	2017	Pakistan	NM	Hospital – CC	20–70 y	Both	NM	AMK	345	16.1	11.7
Junaid et al. [77]	2016	Pakistan	NM	Hospital – CC	15–56 y	Both	Both	ELISA	112	17.32	NM
Roomi et al. [78]	2015	Pakistan	NM	Hospital – CS	18–40 y	Both	Both	EIA	88	8.44	0.49
Junaid et al. [79]	2015	Pakistan	NM	Community - CS	15–50 y	Female	Both	EIA	215	16.6	13.8
Sheikh et al. [80]	2012	Pakistan	Urban	Community - CS	30–80 y	Both	Both	RIA	300	18.8	NM
Mehboobali et al. [81]	2015	Pakistan	NM	Community - CS	18–60 y	Both	Lower	ECLIA	858	20.63	7.25
Mansoor et al. [82]	2010	Pakistan	Urban	Hospital – CS	20–75 y	Both	NM	RIA	123	16.44	3.78
Mustafa et al. [83]	2018	Pakistan	Both	Hospital – CS	18–37 y	Female	Both	CMIA	67	8.348	3.076
Rehman et al. [28]	2018	Pakistan	NM	Hospital – CS	25–55 y	Male	NM	NM	313	30.426	5.83
Khan et al. [84]	2019	Pakistan	Urban	Hospital – CS	Median age 20 y	Both	NM	CLIA	167	12.1	8.81
Nadeem et al. [29]	2018	Pakistan	NM	Community - CS	19–25 y	Both	NM	NM	221	9.8	8.7
Iqbal et al. [85]	2019	Pakistan	NM	Hospital – CS	19–69 y	Both	Both	CK	226	15	10.7
Mahmood et al. [86]	2009	Pakistan	NM	Hospital – CS	16–62 y	Both	NM	ECLIA	244	15.65	9.91
Afsar et al. [30]	2014	Pakistan	NM	Hospital – CS	16–80 y	Both	NM	NM	376	22.11	8.56
Kumar et al. [31]	2016	Pakistan	NM	Hospital – CS	Mean age 40.2 y	Both	NM	NM	160	32	6.6
Sharif et al. [87]	2013	Pakistan	NM	Hospital – CC	20–40 y	Female	Both	ELISA	60	11.04	8.44
Khan et al. [88]	2012	Pakistan	Both	Community - CS	18 y or above	Female	Both	ECLIA	305	8.708	8.664
Dar et al. [89]	2012	Pakistan	Urban	Community - CS	18–48 y	Female	NM	ECLIA	174	15.32	6.16
Meyer et al. [90]	2007	Sri Lanka	Urban	Community - CS	30–60 y	Both	NM	RIA	196	21.68	NM
Haugen et al. [91]	2016	Nepal	Urban	Community - CS	17–44 y	Female	NM	LCMS	500	18.96	6.56
Sherchand et al. [92]	2018	Nepal	NM	Hospital – CS	18 y or above	Both	NM	CLIA	300	19	NM

Abbreviations: *RCT* Randomized Control Trial, *CC* Case Control, *CS* Cross Sectional, *RIA* Radio Immune assay, *LCMS* Liquid chromatography tandem mass spectroscopy, *CLIA* Chemiluminiscence immunoassay, *CMIA* Chemiluminiscent micro particle immune assay, *EIA* Enzyme Immunoassay, *RAIA* Random access Immunoassay, *AMK* Automated Kit, *ELISA* Enzyme Linked Immune Sorbent Assay, *ECLIA* Electrochemiluminiscence Immunoassay, Commercial *Kits* CK, *NM* Not Mention, *y* Year

The total population size of the studies finally selected was 44,717; which included both men and women. Participants were 18 years or above for maximum number of the studies. However, seven studies included adult participants whose age range started from below 18 years [30, 47, 74, 75, 77, 79, 86].

Prevalence of vitamin D deficiency and average level of vitamin D was mentioned in all studies. Prevalence of vitamin D deficiency ranged from 17 to 99% and the average vitamin D level ranged from 4.7 ng/mL to 32 ng/mL. The overall pooled prevalence of vitamin D deficiency was 68% [95% CI: 64 to 72%] and the weighted mean level and weighted standard deviation (weighted SD) of vitamin D was 19.15 ng/mL and 11.59 ng/mL respectively.

**Fig. 2:**
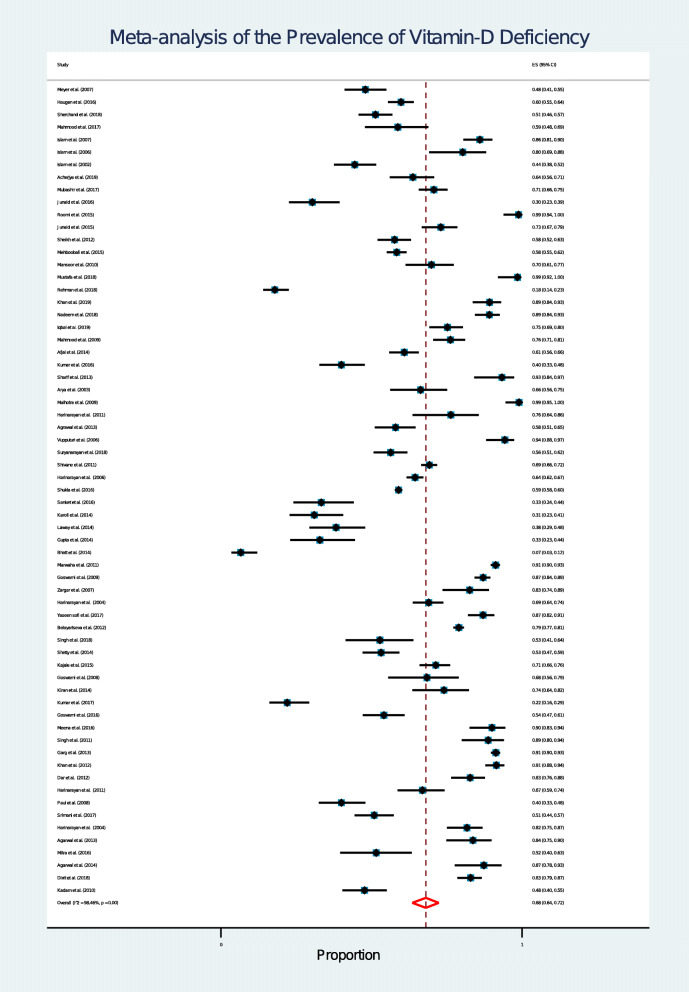
Forest plot represent overall pooled prevalence of vitamin D deficiency among South Asian adults Each horizontal line of the forest plot represents an individual study and the box plotted as prevalence for that study. Diamond at the bottom represent overall prevalence when all the individual studies are combined together and averaged. The horizontal points of the diamond represent the limit of 95% confidence interval

There was a significant amount of heterogeneity in the prevalence of vitamin D deficiency (I^2^ = 98.46%; *p* = 0.00). Forest plot shows overall prevalence of vitamin D deficiency (Fig. 2).

Approximately 95% (62 out of 65) of our selected studies were conducted on the population of the Indian sub-continent (Bangladesh, India and Pakistan). Bar diagram (Additional file; Figure: A1) showed that weighted mean level of vitamin D was less than 20 ng/mL for this region.

### Analysis according to country

**Fig. 3:**
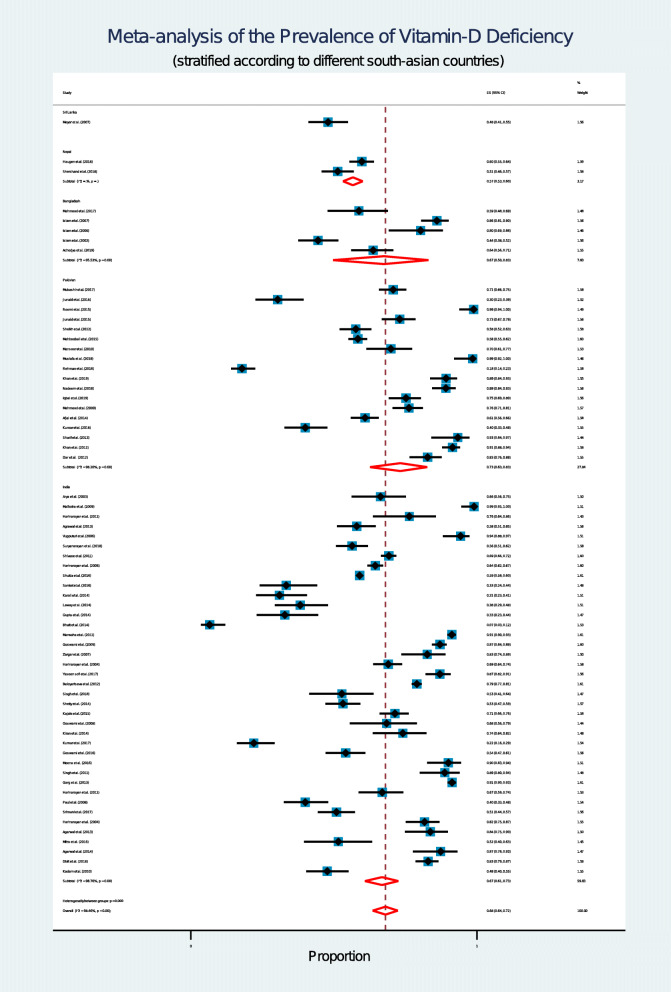
Forest plot shows country-wise prevalence of vitamin D deficiency among South Asian adults In this forest plot all the diamonds except the last one represents polled prevalence in accordance with country; Sri Lanka (first), Nepal (second), Bangladesh (third), Pakistan (fourth) and India (fifth). Each horizontal line of the forest plot represents an individual study and the box plotted as prevalence for that study. The horizontal points of the diamond represent the limit of 95% confidence interval

The study encompassed 5 out of 8 SA countries which included Bangladesh, India, Pakistan Nepal and Sri Lanka. No studies were found from Bhutan, Afghanistan and Maldives regarding vitamin D status. A forest plot displays country wise prevalence of vitamin D deficiency (Fig. 3).

### India

We found 39 studies from India which consisted of 38,672 participants. Mostly, these were cross sectional studies, which were either hospital or community based. Only four of these studies were case control and two were randomized control trial [32–70].

Out of 39 studies, 17 studies were conducted among urban populations, 3 studies on the rural population and 6 studies were conducted on population from both rural and urban areas. The remaining 13 studies did not mention demographic areas for the study population. The majority of the studies did not mention socio-economic status for the study population. The weighted mean level of vitamin D for study participants was 19.34 ng/mL (weighted SD 12.08 ng/mL) [Mean vitamin D level ranged from 4.7 ng/mL to 30.6 ng/mL]. Random effect meta-analysis showed that the weighted pooled prevalence of vitamin D deficiency was 67% [95% CI: 61 to 73%]. This finding indicated ahigh degree of heterogeneity among the population. (I^2^ = 98.76%; *p* = 0.00).

### Bangladesh

We found 5 studies from Bangladesh which consisted of 695 participants. These were either cross sectional or case control studies, being hospital or community based. Out of 5 studies, 2 studies were conducted among urban populations and the other 3 studies were on both rural and urban populations in Bangladesh. Most of the study participants belong to the lower socioeconomic class [71–75]. The weighted mean level of vitamin D for study participants was 16.14296 ng/mL (weighted SD 4.83 ng/mL) [Mean vitamin D level ranged from 12.3 ng/mL to 18.6 ng/mL] and random effect meta-analysis showed that the weighted pooled prevalence of vitamin D deficiency was 67% [95% CI: 50 to 83%]. A significant amount of heterogeneity was present (I^2^ = 95.53%; *p* = 0.00).

### Pakistan

We found 18 studies from Pakistan which consisted of 4354 participants. Study setting was either hospital based or community based and study design was cross sectional for most of the studies. Most of the studies did not mention demographic areas (urban/rural) for study population, 4 studies were conducted among urban populations and two studies included both rural and urban populations. Socio economic status for study participants was not mentioned in most of the studies, 8 studies were conducted on both lower and upper socio-economic groups of population and only one study was conducted on lower class population [28–31, 76–89]. The weighted mean level of vitamin D for study participants was 17.93ng/mL (weighted SD 8.24 ng/mL) [Mean vitamin D level ranged from 8.44 ng/mL to 32ng/mL] and random effect meta-analysis showed that the weighted pooled prevalence of vitamin D deficiency was 73% [95% CI: 63 to 83%] with high degree of heterogeneity (I^2^ = 98.20%; *p* = 0.00).

### Sri Lanka

We found only one study from Sri Lanka which was a community based cross-sectional study. Socioeconomic status was not mentioned. There were 196 participants and among them 47.95% were vitamin D deficient with mean vitamin D level of 21.68 ng/mL [90].

### Nepal

We found 2 studies from Nepal consisting of 800 participants together. Out of 2 studies, one study mentioned demographic area for study population but socioeconomic status was not mentioned for any of these studies [91, 92]. Study setting was either hospital based or community based and study design was cross sectional. Random effect meta-analysis showed that 57% [95% CI: 53 to 60%] of participants were vitamin D deficient with 19 ng/mL mean vitamin D level.

### Analysis according to gender

**Fig. 4:**
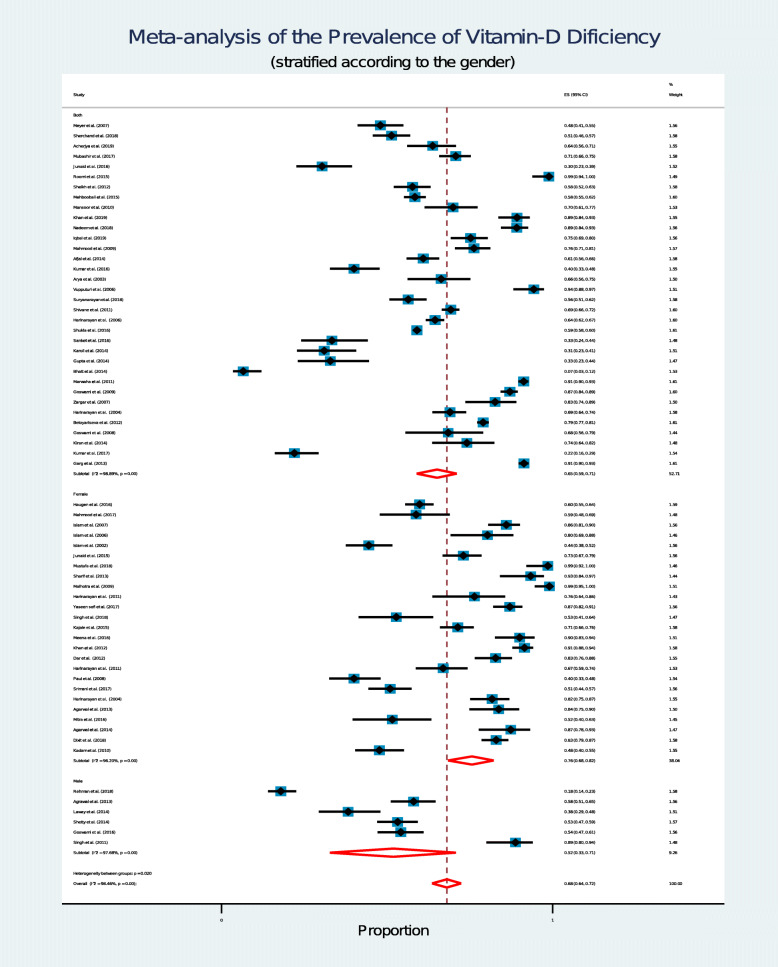
Forest plot shows gender wise prevalence of vitamin D deficiency among South Asian adult population Each horizontal line of the forest plot represents an individual study and the box plotted as prevalence for that study. In this forest plot all the diamonds except the last one represents polled prevalence in accordance with gender; studies which represent participants from both gender (first), female (second) and male (third). The horizontal points of the diamond represent the limit of 95% confidence interval

Out of 65 studies, 25 studies were conducted on adult females and 6 studies were conducted on adult males. Rest of the studies included both male and female adults as their participants. Gender-wise Forest plot is available in Fig. 4.

### Studies including participants from both gender

We found 35 studies which included participants from both gender (male and female). Among these studies, 20 studies were conducted on Indians, 12 studies on Pakistanis and a single study on Bangladeshi, Nepali and Sri Lankan adult. These studies comprised of 39,566 participants together and random effect meta-analysis showed that 65% [95% CI: 59 to 71%] of them were vitamin D deficient with a high degree of heterogeneity (I^2^ = 98.89%; *p* = 0.00). Average vitamin D level of study participants ranged from 7 to 32 ng/mL [Weighted mean 19.54 ng/mL and weighted SD 12.06 ng/mL].

### Studies including only female adults

We found 25 studies which included only female adults as participants. Among these studies,15 was conducted on Indians, 5 on Pakistanis, 4 on Bangladeshis and only one study on Nepali adult females. Together, these studies consisted of 4112 participants and random effect meta-analysis showed that 76% [95% CI: 68 to 82%] of study participants were vitamin D deficient with high number of heterogeneity (I^2^ = 96.20%; *p* = 0.00). The weighted mean vitamin D level of study participants was 14.68ng/mL (weighted SD 7.86 ng/mL).

### Studies including only male adults

We found five studies which included only adult males as participants and all of these studies were conducted on Indian males. These studies comprised of 1039 participants and random effect meta-analysis showed that 51% [95% CI: 33 to 71%] of study participants were vitamin D deficient with high number of heterogeneity (I^2^ = 97.68%; *p* = 0.00). Weighted mean vitamin D level of study participants was 22.13 ng/mL (weighted SD 7.39 ng/mL).

A bar diagram shows weighted mean level of vitamin D among SA male and female (Additional file; Fig. A2).

### Analysis according to time

**Fig. 5:**
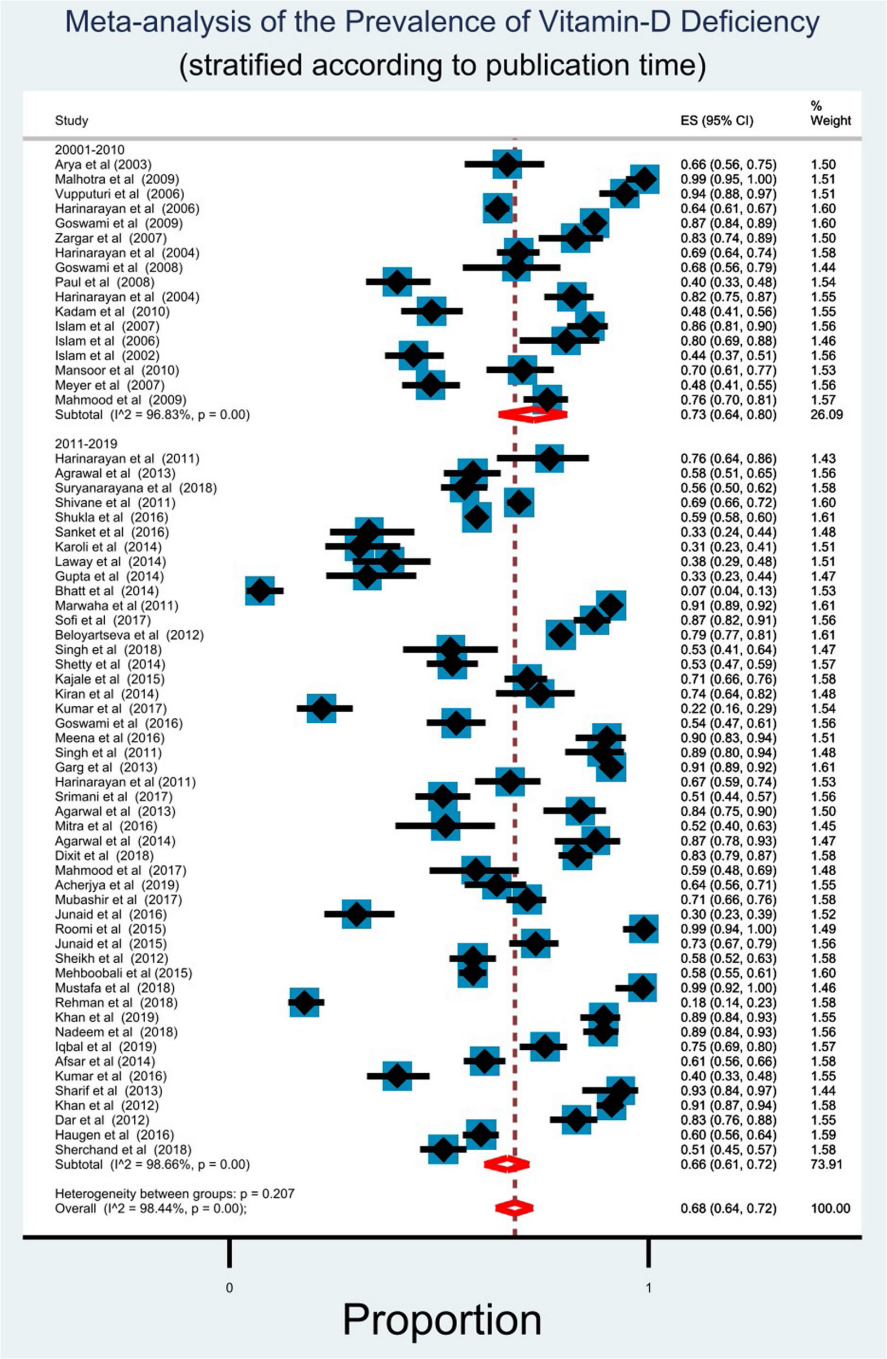
Forest plot shows prevalence of vitamin D deficiency in accordane with time among south Asian adult population. Each horizontal line of the forest plot represents an individual study and the box plotted as prevalence for that study. In this forest plot all the diamonds except the last one represents polled prevalence in accordance with time; studies which conducted between 2001 and 2010 (first) and 2011–2020 (second). The horizontal points of the diamond represent the limit of 95% confidence interval

We segregated our selected studies into two groups; (1) studies conducted between 2001 and 2010, and (2) those conducted between 2011 and 2019. We found that most of the studies (48 out of 65) were conducted in the last decade (2011–2019). Random effect meta-analysis showed that prevalence of vitamin D deficiency was higher in 2001–2010 (73%; 95% CI: 64–80%) in comparison with 2011–2019 (66%; 95% CI: 61–72%). However, high degree of heterogeneity was observed in both time frame (I^2^ value of 96.83 and 98.66% for the two decades respectively; *p* = 0.00). Forest plot for this comparison is available in Fig. 5.

### Quality assessment

Risk of bias score was calculated for each of the studies (Additional fie: Table A2) following the method described by Hoy et al. in the year 2012 [23]. Studies that scored between 0 and 3 can be considered as having low risk of bias, and studies that scored 4–6 are moderate risk, and studies with scores of 7–9 can be considered as having high risk of bias. Among the 65 studies we selected; no study was found to contain a high risk of bias. Twenty-two studies showed low risk of bias, while the rest had a moderate risk of bias.

### Publication bias

The funnel plot for the prevalence of vitamin D deficiency is presented in the Additional fie which indicated the existence of asymmetry and publication bias (Additional fie; Fig. A3). The Eggers test was found to be statistically insignificant which suggested no small study effects (*p* = 0.921) among the studies.

In this systematic review and meta-analysis, we calculated the overall pooled prevalence of vitamin D deficiency among SA adults. We also showed country-wise and gender-wise prevalence of vitamin D deficiency in our sub-group analyses. Our findings also indicated that most of the studies were conducted on the Indian population. However, we did not find any studies regarding the vitamin D prevalence in Afghanistan, Maldives and Bhutan.

## Discussion

This study reveals that 68% [95% CI: 64 to 72%] of the SA adults are affected with vitamin D deficiency (Fig. 2). Comparison of this deficiency to the other parts of the world implies that this problem might be worse in SA compared to Southeast Asia (where the prevalence of vitamin D deficiency was found to vary from 6 to 70%) and Europe (around 40%) [93, 94]. We hypothesize that this high prevalence of vitamin D deficiency could be linked to the high prevalence of many health issues in this region. Indeed, this hypothesis is supported by some literature that several communicable and non-communicable diseases are rising in SA [16–18, 95, 96].

While high vitamin D deficiency in SA was the highlight of this study, we also found high degree of heterogeneity in the overall results (I^2^ = 98.46%) (Fig. 2). We assume that geographical location and gender might act as a confounding variable for such a large-scale heterogeneity.

Country-wise comparison showed that Sri Lanka had the lowest percentage (48%) of prevalence, while Pakistan had the highest (73%). Compared to the Pakistani population, Bangladeshi and Indian population had a lower prevalence (67%). It is hypothesized that variability in the degree of sunlight exposure due to difference in geographical locations could be a probable reason for this [11, 97].

The people living in the tropical regions are exposed to more sunlight throughout the year as compared to those who live in subtropical regions. Vitamin D is synthesized naturally in the human body when UV-B from sunlight penetrates our skin and initiates the physiological processes of vitamin D synthesis [1, 3]. Pakistan is located in a sub-tropical region where sunlight availability is relatively low [98]. However, Bangladesh and India both share tropical and subtropical regions in their national map. High prevalence of vitamin D deficiency was also observed in other countries like Iran (56%) and China (70%) which share similar kinds of geographical location on their national maps [99, 100]. This hypothesis can be further bolstered by the fact that the adults from Sri Lanka (a tropical country) showed the lowest prevalence of vitamin D deficiency among all the SA countries considered in this study.

Gender-wise comparison in our study reveals that females could be affected with vitamin D deficiency more than the males in SA; 76% for females (95% CI: 68 to 82%) as opposed to 51% for males (95% CI: 33 to 71%) (Fig. 4). We hypothesize that this might be related to a cultural aspect of this region; as lesser number of SA women spend their time outdoors which effectively limits their exposure to direct sunlight. In 2020, the World Bank reported that in SA the female labor force participation rate is around 24%, whereas male labor force participation rate is 80% [101]. Besides longer indoor time, clothing practice of women in the SA region could also be another cultural factor that could partially explain higher degree of vitamin-D deficiency among the females. The use of burqas (traditional full-body covering), hijab (Muslim wearing that covers the upper part of the body) and other non-religious body-coverings (traditional clothes) block direct sunlight exposure. These types of clothing practices are very common in many states of India, Pakistan and Bangladesh. A recent media report also suggests that these practices have increased exponentially in this region over the past three decades [102].

This hypothesis on the effects of clothing and staying indoor can be further supported by similar trends being observed in case of the women in the Middle East. World Bank reported in 2020 that female labor participation is around 20% in Middle Eastern countries [103]. More than 60% of Middle Eastern people prefer burqa as appropriate dress for Saudi Arabian women [104]. On top of that, a study report showed that 60% of women from Saudi Arabia living in Riyadh city had vitamin D deficiency [105].

Besides geography, gender, and clothing practices, a number of other factors (such as skin color) have also been reported in literature which can further explain the high heterogeneity that was observed in this study [1–3].

In this regard, in our study, we found that the Nepalese population had lower prevalence of vitamin D deficiency (57% [95% CI: 53 to 60%]) compared to the other countries from the Indian sub-continent (India, Pakistan, and Bangladesh) (Fig. 3). According to the Fitzpatrick scale, most of the Nepalese have lighter skin in comparison to population of the sub-continent, who tend to have a darker skin complexion [106]. Though Nepal is located in a subtropical region [98], because of skin color, Nepalese might need less sunlight exposure in comparison to sub-tropical people for the production of similar level of vitamin D. Therefore, the variation of skin complexion could be a potential determinant of the observed variability of vitamin D deficiency among the SA countries.

High prevalence of vitamin D deficiency and low vitamin D status might act as a factor for the rise of these communicable and non-communicable diseases in this region. However, a recent study found that vitamin D deficiency is also associated with cytokine storm (dramatic immune system over reaction) which causes COVID 19 patients more vulnerable [107]. So, policy makers in this region need to take into account the high prevalence of vitamin D deficiency as well as the heterogeneity that has been identified in this study.

While this study highlights a critical health issue, it has few limitations well. Most of the studies that matched our selection criteria (59out of 65) did not mention the socioeconomic conditions (e.g., income status and urban vs. rural difference). So, we could not test the effects of these factors in this study. Furthermore, we did not find any study from Bhutan, Maldives and Afghanistan. Only one study from Sri Lanka matched our inclusion criteria and two from Nepal. As such, because of insufficient data, we could not find the weighted mean level of vitamin D for these two countries. Another limitation is that a range of different methods were used in different studies to assay vitamin D in the serum. This might have introduced an assay bias to our findings. However, this limitation is inherent for all similar studies and was indeed unavoidable.

The high prevalence of vitamin D deficiency among SA adults is a public health challenge that should be addressed as an emergency, some even argue whether this should be treated as an ongoing pandemic [94]. Therefore, effectiveness of the mitigation strategies would be a critical public health challenge for the governments of SA. However, our search did not reveal any national level nutritional guidelines or policies addressing vitamin D deficiency in SA, except in India [108]. While existing guidelines and policies [108, 109] can be used as reference for the SA countries, we argue that the socio-cultural aspects of the individual countries (e.g., clothing practice, skin complexion, and economic status) also need to be taken into consideration. People, especially the women, who practice heavy clothing and those who spend more time indoors can be encouraged to check their serum level of vitamin D on a regular basis and take necessary supplements. For people without adequate sunlight exposure, it has been recommended that, an adult should take 800–1000 IU of vitamin D per day [3]. In addition, SA population in general and especially the women can be encouraged to spend more time to boost natural production of vitamin D synthesis in their body [3]. In this regard, negative attitude towards sunlight exposure can be a big challenge in SA. It has been reported that lack of knowledge about vitamin D and negative attitude towards sunlight exposure is prevalent among Indian and Pakistani students [110, 111]. This lack of knowledge and negative attitude could be a key reason behind staying away from sunlight which can contribute to reduce serum vitamin D level. Introducing mass campaigns about the relationship between sunlight exposure and vitamin D deficiency may be really effective. Moreover, active measures should be taken to increase the number of diagnostic tests for detecting the serum vitamin D level. To achieve this, increasing the number of test centers, reduce price for testing the serum level of vitamin D by providing subsidies can also be considered by the governments in SA region.

## Conclusions

To the best of our knowledge, this is the first systematic review and meta-analysis on the prevalence of vitamin D deficiency among SA adults. Our findings point out that nearly seven out of ten adults in this region are suffering with vitamin D deficiency, while high inter-country variation was revealed. The results have generated evidence that underscores urgency of prioritizing the mitigation strategies. While this systematic review focused on SA only, the knowledge and insight generated from this study are transferable to other regions and countries with comparable geographic and socio-cultural aspects.

## Supplementary Information

**Additional file 1.**

## Data Availability

Only aggregated summaries of the data are provided in this manuscript. However, all data generated in this study can be made publicly available on request. Please contact the corresponding author for any kind of data request.

## References

[1] Nair R, Maseeh A (2012). Vitamin D: The sunshine vitamin. J PharmacolPharmacother.

[2] Mostafa WZ, Hegazy RA (2015). Vitamin D and the skin: Focus on a complex relationship: A review. J Adv Res.

[3] Holick MF (2007). Vitamin D deficiency. New Engl J Med.

[4] Huang SJ, Wang XH, Liu ZD, Cao WL, Han Y, Ma AG, Xu SF (2017). Vitamin D deficiency and the risk of tuberculosis: A meta-analysis. Drug Des DevelTher.

[5] Amrein K, Scherkl M, Hoffmann M et al. Vitamin D deficiency 2.0: an update on the current status worldwide. Eur J ClinNutr. 2020;74(11):1498–513. 10.1038/s41430-020-0558-y. 10.1038/s41430-020-0558-y PMC7091696 31959942

[6] Lips P, Cashman KD, Lamberg-Allardt C, Bischoff-Ferrari HA, Obermayer-Pietsch B, Bianchi ML, Stepan J, el-Hajj Fuleihan G, Bouillon R (2019). Current vitamin D status in European and Middle East countries and strategies to prevent vitamin D deficiency: a position statement of the European Calcified Tissue Society. Eur J Endocrinol.

[7] Kaykhaei MA, Hashemi M, Narouie B, Shikhzadeh A, Rashidi H, Moulaei N, Ghavami S (2011). High prevalence of vitamin D deficiency in Zahedan. Southeast Iran Ann NutrMetab.

[8] Sayed-Hassan R, Abazid N, Alourfi Z (2014). Relationship between 25-hydroxyvitamin D concentrations, serum calcium, and parathyroid hormone in apparently healthy Syrian people. Arch Osteoporos.

[9] Cashman KD, Dowling KG, Škrabáková Z, Kiely M, Lamberg-Allardt C, Durazo-Arvizu RA, Sempos CT, Koskinen S, Lundqvist A, Sundvall J, Linneberg A, Thuesen B, Husemoen LLN, Meyer HE, Holvik K, Grønborg IM, Tetens I, Andersen R (2015). Standardizing serum 25-hydroxyvitamin D data from four Nordic population samples using the Vitamin D Standardization Program protocols: Shedding new light on vitamin D status in Nordic individuals. Scand J Clin Lab Invest.

[10] Souberbielle JC, Massart C, Brailly-Tabard S, Cavalier E, Chanson P (2016). Prevalence and determinants of vitamin D deficiency in healthy French adults: the VARIETE study. Endocrine..

[11] Yeum KJ, Song BC, Joo NS (2016). Impact of geographic location on vitamin D status and bone mineral density. Int J Environ Res and Public Health.

[12] The World Bank. In: South Asia; 2020. https://www.worldbank.org/en/region/sar/overview (Accessed December 18, 2020).

[13] The World Bank. Total population data. 2019. https://data.worldbank.org/indicator/SP.POP.TOTL (Accessed December 18, 2020).

[14] Ryabchikov, AleksandrMaximovich, Sivaramamurti, Calambur,Yefremov, YuryKonstantinovich and Alexeeva, Nina Nikolaevna. "South Asia". Encyclopedia Britannica, 1 Sep. 2020, https://www.britannica.com/place/South-Asia. (Accessed December 2020).

[15] Barai MK, South Asia (2015). South Asia in the 21st Century Global Order: Problems, Promises and Positioning. Focus J Int Bus.

[16] Siegel KR, Patel SA, Ali MK (2014). Non-communicable diseases in South Asia: contemporary perspectives. Br Med Bull.

[17] Laxminarayan R, Kakkar M, Horby P, Malavige GN, Basnyat B (2017). Emerging and re-emerging infectious disease threats in South Asia: status, vulnerability, preparedness, and outlook. BMJ..

[18] Zaidi AKM, Awasthi S, DeSilva HJ (2004). Burden of infectious diseases in South Asia. Br Med J.

[19] Selvarajan S, Gunaseelan V, Anandabaskar N, Xavier AS, Srinivasamurthy S, Kamalanathan SK, Sahoo JP (2017). Systematic review on vitamin D level in apparently healthy Indian population and analysis of its associated factors. Ind J EndocrinolMetab.

[20] Moher D, Shamseer L, Clarke M (2015). Preferred reporting items for systematic review and meta-analysis protocols (PRISMA-P) 2015 statement. Syst Rev.

[21] Holick MF, Binkley NC, Bischoff-Ferrari HA, Gordon CM, Hanley DA, Heaney RP, Murad MH, Weaver CM, Endocrine Society (2011). Evaluation, treatment, and prevention of vitamin D deficiency: An endocrine society clinical practice guideline. J Clin Endocrinol Metab.

[22] Jeyakumar A, Shinde V (2019). A systematic review and meta-analysis of prevalence of vitamin D deficiency among adolescent girls in selected Indian states. Nutr Health.

[23] Hoy D, Brooks P, Woolf A, Blyth F, March L, Bain C, Baker P, Smith E, Buchbinder R (2012). Assessing risk of bias in prevalence studies: Modification of an existing tool and evidence of interrater agreement. J ClinEpidemiol.

[24] Chowdhury MZI, Rahman M, Akter T, Akhter T, Ahmed A, Shovon MA, Farhana Z, Chowdhury N, Turin TC (2020). Hypertension prevalence and its trend in Bangladesh: evidence from a systematic review and meta-analysis. ClinHypertens..

[25] Nyaga VN, Arbyn M, Aerts M (2014). Metaprop: a Stata command to perform meta-analysis of binomial data. Arch Public Health.

[26] Chowdhury MZI, Anik AM, Farhana Z (2018). Prevalence of metabolic syndrome in Bangladesh: a systematic review and meta-analysis of the studies. BMC Public Health.

[27] Higgins JPT, Thompson SG, Deeks JJ, Altman DG (2003). Measuring inconsistency in meta-analyses. Br Med J.

[28] Rehman R, Lalani S, Baig M, Nizami I, Rana Z, Gazzaz ZJ (2018). Association between vitamin D, reproductive hormones and sperm parameters in infertile male subjects. Front Endocrinol.

[29] Nadeem S, Munim TF, Hussain HF, Hussain DF (2018). Determinants of vitamin D deficiency in asymptomatic healthy young medical students. Pak J Med Sci.

[30] Afsar SS, Idrees M, Gulzar M (2014). Vitamin D (25-OH) levels in asymptomatic healthy population. Rawal Medical Journal.

[31] Kumar S, Almani Z, Almani SA, Nazia S (2016). Relation of vitamin D level with demographic and lab feature for local population. J Liaquat Uni Med Health Sci.

[32] Arya V, Bhambri R, Godbole MM, Mithal A (2004). Vitamin D status and its relationship with bone mineral density in healthy Asian Indians. Osteoporos Int.

[33] Malhotra N, Mithal A, Gupta S, Shukla M, Godbole M (2009). Effect of vitamin D supplementation on bone health parameters of healthy young Indian women. Arch Osteoporos.

[34] Harinarayan CV, Sachan A, Reddy PA, Satish KM, Prasad UV, Srivani P (2011). Vitamin D status and bone mineral density in women of reproductive and postmenopausal age groups: a cross-sectional study from south India. J Assoc Physicians India.

[35] Agrawal NK, Sharma B (2013). Prevalence of osteoporosis in otherwise healthy Indian males aged 50 years and above. Arch Osteoporos.

[36] Rao Vupputuri M, Goswami R, Gupta N, Ray D, Tandon N, Kumar N (2006). Prevalence and functional significance of 25-hydroxyvitamin D deficiency and vitamin D receptor gene polymorphisms in Asian Indians. Am J ClinNutr.

[37] Suryanarayana P, Arlappa N, Santhosh VS (2018). Prevalence of vitamin D deficiency and its associated factors among the urban elderly population in Hyderabad metropolitan city. South India Ann Hum Biol.

[38] Shivane VK, Sarathi V, Bandgar T, Menon P, Shah NS (2011). High prevalence of hypovitaminosis D in young healthy adults from the western part of India. Postgrad Med J.

[39] Harinarayan CV, Ramalakshmi T, Prasad UV, Sudhakar D, Srinivasarao PV, Sarma KV, Kumar EG (2007). High prevalence of low dietary calcium, high phytate consumption, and vitamin D deficiency in healthy south Indians. Am J Clin Nutr.

[40] Shukla K, Sharma S, Gupta A, Raizada A, Vinayak K (2016). Current scenario of prevalence of vitamin D deficiency in ostensibly healthy Indian population: A hospital based retrospective study. Ind J ClinBiochem.

[41] Sanket S, Madireddi J, Stanley W, Sura P, Prabhu M (2016). Relation between vitamin D deficiency and severity of chronic obstructive pulmonary disease-A case control study. J ClinDiagn Res.

[42] Karoli R, Fatima J, Gupta SS, Shukla V (2015). Moidurrehman, Manhar M. Vitamin D deficiency in medical patients at a teaching hospital in North India. J Assoc of Physicians India.

[43] Laway BA, Kotwal SK, Shah ZA (2014). Pattern of 25 hydroxy vitamin D status in North Indian people with newly detected type 2 diabetes: A prospective case control study. Indian J EndocrinolMetab..

[44] Gupta A, Prabhakar S, Modi M, Bhadada SK, Lal V, Khurana D (2014). Vitamin D status and risk of ischemic stroke in North Indian patients. Indian J EndocrinolMetab.

[45] Bhatt SP, Misra A, Sharma M, Guleria R, Pandey RM, Luthra K, Vikram NK (2014). Vitamin D insufficiency is associated with abdominal obesity in urban Asian Indians without diabetes in North India. Diabetes TechnolTher.

[46] Marwaha RK, Tandon N, Garg MK, Kanwar R, Narang A, Sastry A, Saberwal A, Bandra K (2011). Vitamin D status in healthy Indians aged 50 years and above. J Assoc Physicians India.

[47] Goswami R, Marwaha RK, Gupta N, Tandon N, Sreenivas V, Tomar N, Ray D, Kanwar R, Agarwal R (2009). Prevalence of vitamin D deficiency and its relationship with thyroid autoimmunity in Asian Indians: a community-based survey. Br J Nutr.

[48] Zargar AH, Ahmad S, Masoodi SR, Wani AI, Bashir MI, Laway BA, Shah ZA (2007). Vitamin D status in apparently healthy adults in Kashmir Valley of Indian subcontinent. Postgrad Med J.

[49] Harinarayan CV, Ramalakshmi T, Venkataprasad U (2004). High prevalence of low dietary calcium and low vitamin D status in healthy south Indians. Asia Pac J ClinNutr.

[50] Sofi NY, Jain M, Kapil U, Seenu V, Ramakrishnan L, Yadav CP, Pandey RM (2017). Status of serum vitamin D and calcium levels in women of reproductive age in national capital territory of India. Indian J EndocrinolMetab..

[51] Beloyartseva M, Mithal A, Kaur P, Kalra S, Baruah MP, Mukhopadhyay S, Bantwal G, Bandgar TR (2012). Widespread vitamin D deficiency among Indian health care professionals. Arch Osteoporos.

[52] Singh V, Barik A, Imam N (2019). Vitamin D3 Level in Women with Uterine Fibroid: An Observational Study in Eastern Indian Population. J ObstetGynaecol India.

[53] Shetty S, Kapoor N, Naik D, Asha HS, Prabu S, Thomas N, Seshadri MS, Paul TV (2014). Osteoporosis in healthy South Indian males and the influence of life style factors and vitamin D status on bone mineral density. JOsteoporos..

[54] Kajale N, Khadilkar A, Chiplonkar S (2016). A Cross-Sectional Study of Postpartum Changes in Bone Status in Indian Mothers. J ObstetGynecol India.

[55] Goswami R, Kochupillai N, Gupta N, Goswami D, Singh N, Dudha A (2008). Presence of 25 (OH) D deficiency in a rural North Indian village despite abundant sunshine. J Assoc Physicians India.

[56] Kiran B, Prema A, Thilagavathi R, Rani RJ (2014). Serum 25-Hydroxy vitamin D, calcium, phosphorus and alkaline phosphatase levels in healthy adults above the age of 20 living in Potheri Village of Kancheepuram District. Tamilnadu J App Pharm Sci.

[57] Kumar R, Kumar P, Saxena KN, Mishra M, Mishra VK, Kumari A, Dwivedi M, Misra SP (2017). Vitamin D status in patients with cirrhosis of the liver and their relatives—A case control study from North India. Indian J Gastroenterol.

[58] Goswami R, Saha S, Sreenivas V, Singh N (2017). Vitamin D - binding protein, vitamin D status and serum bioavailable 25 (OH) D of young Asian Indian males working in outdoor and indoor environments. J Bone Miner Metab.

[59] Meena P, Dabas A, Shah D, Malhotra RK, Madhu SV, Gupta P (2017). Sunlight exposure and vitamin D status in breastfed infants. Indian Pediatr.

[60] Singh SK, Prakash V, Tiwari S, Daliparthy DP, Singh S, Jain P (2011). Summer and winter prevalence of vitamin D deficiency of young resident doctors in North India. Nutrition & Dietetics.

[61] Garg MK, Tandon N, Marwaha RK, Menon AS, Mahalle N (2014). The relationship between serum 25-hydroxy vitamin D, parathormone and bone mineral density in Indian population. ClinEndocrinol..

[62] Harinarayan CV, Sachan A, Reddy PA, Satish KM, Prasad UV, Srivani P (2011). Vitamin D status and bone mineral density in women of reproductive and postmenopausal age groups: a cross-sectional study from south India. JAPI..

[63] Paul TV, Thomas N, Seshadri MS, Oommen R, Jose A, Mahendri NV (2008). Prevalence of osteoporosis in ambulatory postmenopausal women from a semiurban region in Southern India: relationship to calcium nutrition and vitamin D status. EndocrPract.

[64] Srimani S, Saha I, Chaudhuri D (2017). Prevalence and association of metabolic syndrome and vitamin D deficiency among postmenopausal women in a rural block of West Bengal. India PloS One.

[65] Harinarayan CV (2005). Prevalence of vitamin D insufficiency in postmenopausal south Indian women. Osteoporos Int.

[66] Agarwal N, Mithal A, Dhingra V, Kaur P, Godbole MM, Shukla M (2013). Effect of two different doses of oral cholecalciferol supplementation on serum 25-hydroxy-vitamin D levels in healthy Indian postmenopausal women: A randomized controlled trial. Ind J EndocrinolMetab.

[67] Mitra S, Nayak PK, Agrawal S, Sahoo JP, Kamalanathan S, Nanda R (2016). Vitamin D status and cardio-metabolic risk in Indian postmenopausal women. J ClinDiag Res.

[68] Agarwal N, Mithal A, Kaur P, Dhingra V, Godbole MM, Shukla M (2014). Vitamin D and insulin resistance in postmenopausal Indian women. Indian J EndocrinolMetab..

[69] Dixit V, Tripathi RL, Dhanwal DK (2018). All 25-hydroxyvitamin D-deficient Indian postmenopausal women do not have secondary hyperparathyroidism. Arch Osteoporos.

[70] Kadam N, Chiplonkar S, Khadilkar A, Divate U, Khadilkar V (2010). Low bone mass in urban Indian women above 40 years of age: prevalence and risk factors. GynecolEndocrinol..

[71] Mahmood S, Rahman M, Biswas SK, Saqueeb SN, Zaman S, Manirujjaman M, Perveen R, Ali N (2017). Vitamin D and Parathyroid Hormone Status in Female Garment Workers: A Case-Control Study in Bangladesh. Biomed Res Int.

[72] Islam Z, Shamim AA, Kemi V (2008). Vitamin D deficiency and low bone status in adult female garment factory workers in Bangladesh. Br J Nutr.

[73] Islam MZ, Akhtaruzzaman M, Lamberg-Allardt C (2006). Hypovitaminosis D is common in both veiled and non-veiled Bangladeshi women. Asia Pac J ClinNutr.

[74] Islam MZ, Lamberg-Allardt C, Kärkkäinen M, Outila T, Salamatullah Q, Shamim AA (2002). Vitamin D deficiency: a concern in premenopausal Bangladeshi women of two socio-economic groups in rural and urban region. Eur J ClinNutr..

[75] Acherjya GK, Ali M, Tarafder K, Akhter N, Chowdhury MK, Islam DU, Rahman MH, Miah MT (2019). Study of vitamin D deficiency among the apparently healthy population in Jashore. Bangladesh Mymensingh Med J.

[76] Mubashir M, Anwar S, Tareen AK, Mehboobali N, Iqbal K, Iqbal MP (2017). Association of vitamin D deficiency and VDBP gene polymorphism with the risk of AMI in a Pakistani population. Pak J Med Sci.

[77] Junaid K, Rehman A, Jolliffe DA, Saeed T, Wood K, Martineau AR (2016). Vitamin D deficiency associates with susceptibility to tuberculosis in Pakistan, but polymorphisms in VDR, DBP and CYP2R1 do not. BMC Pulm Med.

[78] Roomi MA, Farooq A, Ullah E, Lone KP (2015). Hypovitaminosis D and its association with lifestyle factors. Pak J Med Sci.

[79] Junaid K, Rehman A, Jolliffe DA, Wood K, Martineau AR (2015). High prevalence of vitamin D deficiency among women of child-bearing age in Lahore Pakistan, associating with lack of sun exposure and illiteracy. BMC Womens Health.

[80] Sheikh A, Saeed Z, Jafri SA, Yazdani I, Hussain SA (2012). Vitamin D levels in asymptomatic adults-a population survey in Karachi. Pakistan PloS One.

[81] Mehboobali N, Iqbal SP, Iqbal MP (2015). High prevalence of vitamin D deficiency and insufficiency in a low-income peri-urban community in Karachi. J Pak Med Assoc.

[82] Mansoor S, Habib A, Ghani F, Fatmi Z, Badruddin S, Mansoor S, Siddiqui I, Jabbar A (2010). Prevalence and significance of vitamin D deficiency and insufficiency among apparently healthy adults. ClinBiochem..

[83] Mustafa G, Asadi MA, Iqbal I, Bashir N (2018). Low vitamin D status in nursing Pakistani mothers in an environment of ample sunshine: a cross-sectional study. BMC Pregnancy Childbirth.

[84] Khan AH, Fatima SS, Raheem A, Jafri L (2019). Are serum leptin levels predicted by lipoproteins, vitamin D and body composition?. World J Diabetes.

[85] Iqbal K, Islam N, Mehboobali N, Asghar A, Iqbal SP, Iqbal MP (2019). Relationship of sociodemographic factors with serum levels of vitamin D in a healthy population of Pakistan. Pak J Pharm Sci.

[86] Mahmood K, Akhtar ST, Talib A, Haider I (2009). Vitamin-D status in a population of healthy adults in Pakistan. Pak J Med Sci.

[87] Sharif S, Farasat T, Shoaib H, Saqib M, Fazal S (2013). Vitamin D levels among pregnant and lactating women. J Coll Physicians Surg Pak.

[88] Khan AH, Iqbal R, Naureen G, Dar FJ, Ahmed FN (2012). Prevalence of vitamin D deficiency and its correlates: results of a community-based study conducted in Karachi. Pakistan Arch Osteoporos.

[89] Dar FJ, Iqbal R, Ghani F, Siddiqui I, Khan AH (2012). Bone health status of premenopausal healthy adult females in Pakistani females. Arch Osteoporos.

[90] Meyer HE, Holvik K, Lofthus CM, Tennakoon SU (2008). Vitamin D status in Sri Lankans living in Sri Lanka and Norway. Br J Nutr.

[91] Haugen J, Ulak M, Chandyo RK, Henjum S, Thorne-Lyman AL, Ueland PM, Midtun Ø, Shrestha P, Strand T (2016). Low prevalence of vitamin D insufficiency among Nepalese infants despite high prevalence of vitamin D insufficiency among their mothers. Nutrients..

[92] Sherchand O, Sapkota N, Chaudhari RK, Khan SA, Baranwal JK, Pokhrel T, Das BK, Lamsal M (2018). Association between vitamin D deficiency and depression in Nepalese population. Psychiatry Res.

[93] Nimitphong H, Holick MF (2013). Vitamin D status and sun exposure in Southeast Asia. Dermatoendocrinol..

[94] Cashman KD, Dowling KG, Škrabáková Z (2016). Vitamin D deficiency in Europe: Pandemic?. Am J ClinNutr..

[95] Maswood MH (2018). Cardiac patients rising in Bangladesh, 2.5 lakh die annually. New Age.

[96] Special Correspondent (2019). 87,090 women die of breast cancer in India every year. The Hindu.

[97] Krzywanski J, Mikulski T, Krysztofiak H, Mlynczak M, Gaczynska E, Ziemba A (2016). Seasonal vitamin D status in Polish elite athletes in relation to sun exposure and oral supplementation. PLoS One.

[98] Mavridou A, Pappa O, Papatzitze O (2018). [MAP]. Exotic tourist destinations and transmission of infections by swimming pools and hot springs —a literature review. Int J Environ Res Public Health.

[99] Vatandost S, Jahani M, Afshari A, Amiri MR, Heidarimoghadam R, Mohammadi Y (2018). Prevalence of vitamin D deficiency in Iran: a systematic review and meta-analysis. Nutr Health.

[100] Wei J, Zhu A, Ji JS (2019). A Comparison Study of Vitamin D Deficiency among Older Adults in China and the United States. Sci Rep.

[101] The World Bank (2020). South Asia. South Asia Women in the Workforce Week.

[102] Shams S (2016). Why wearing the burqa is on the rise in South Asia? DW.

[103] The World Bank (2020). Data. Labor force participation rate, female (% of female population ages 15+) (modeled ILO estimate) - Middle East & North Africa.

[104] Fisher M. This fascinating chart shows how Middle Easterners think women should dress: The Washington Post; 2014. World Views. Accessed December 30. https://www.washingtonpost.com/news/worldviews/wp/2014/01/08/this-fascinating-chart-shows-how-middle-easterners-think-women-should-dress/

[105] AlFaris NA, AlKehayez NM, AlMushawah FI, AlNaeem AN, AlAmri ND, AlMudawah ES (2019). Vitamin D deficiency and associated risk factors in women from Riyadh. Saudi Arabia Sci Rep.

[106] Jha AK, Gurung D (2006). Seasonal variation of skin diseases in Nepal: a hospital based annual study of out-patient visits. Nepal Medical College journal: NMCJ.

[107] Benskin LL (2020). A Basic Review of the Preliminary Evidence That COVID-19 Risk and Severity Is Increased in Vitamin D Deficiency. Front Public Health.

[108] Indian Council of Medical Research, Department of Health Research, Ministry of Health and Family Welfare, Government of India. Summary of recommendations-ICMR-NIN (2020). RDA and EAR-A short report reference body weight.

[109] Aguiar M, Andronis L, Pallan M, Högler W, Frew E (2017). Preventing vitamin D deficiency (VDD): A systematic review of economic evaluations. Eur J Pub Health.

[110] Arora HA, Dixit VI, Srivastava NI (2016). Evaluation of knowledge, practices of vitamin D and attitude toward sunlight among Indian students. Asian J Pharm Clin Res.

[111] Tariq A, Khan SR, Basharat A (2020). Assessment of knowledge, attitudes and practice towards vitamin D among university students in Pakistan. BMC Public Health.

