# Supplementary material for: High prevalence of vitamin D deficiency among the South Asian adults: a systematic review and meta-analysis
Source: BMC Public Health. 2021 Oct 9;21:1823. doi: 10.1186/s12889-021-11888-1 (PMC8501935; doi:10.1186/s12889-021-11888-1)
Supplement: Supplementary file 1 — Additional file 1. [file 12889_2021_11888_MOESM1_ESM.docx]

**Table A1:** Searching detail, MeSH terms and alternative terms used in this study

| **PubMed** | **SCOPUS** | **Google Scholar** |
| --- | --- | --- |
| (“Vitamin D Deficiency”[MeSH]) AND Bangladesh[MeSH] AND healthy adults [MeSH]  (“Vitamin D”[MeSH]) AND Bangladesh[MeSH] AND healthy adults [MeSH]  (“Vitamin D”[MeSH]) AND Bangladesh[MeSH] AND Prevalence [MeSH] AND healthy adults [MeSH]  (“Vitamin D Deficiency”[MeSH]) AND Bangladesh[MeSH] AND young adults[MeSH]  (“Vitamin D”[MeSH]) AND Bangladesh[MeSH] AND Prevalence [MeSH] AND young adults [MeSH]  (“Vitamin D”[MeSH]) AND Bangladesh[MeSH] AND young adults [MeSH]  (“Vitamin D Deficiency”[MeSH]) AND Bangladesh[MeSH] AND adults Males [MeSH]  (“Vitamin D”[MeSH]) AND Bangladesh[MeSH] AND adult Males [MeSH]  (“Vitamin D”[MeSH]) AND Bangladesh[MeSH] AND Prevalence [MeSH] AND adult Males [MeSH]  (“Vitamin D Deficiency”[MeSH]) AND Bangladesh[MeSH] AND adults Females [MeSH]  (“Vitamin D”[MeSH]) AND Bangladesh[MeSH] AND adult Females [MeSH]  (“Vitamin D”[MeSH]) AND Bangladesh[MeSH] AND Prevalence [MeSH] AND adult Females [MeSH]  (“Vitamin D Deficiency”[MeSH]) AND Bangladesh[MeSH] AND Premenopausal women [MeSH]  (“Vitamin D”[MeSH]) AND Bangladesh[MeSH] AND Premenopausal women [MeSH] | (INDEXTERMS(“Vitamin D Deficiency”) AND INDEXTERMS(BANGLADESH) AND INDEXTERMS(Healthy adults)) OR (TITLE-ABS-KEY(“Vitamin D”) AND TITLE-ABS-KEY(BANGLADESH) AND TITLE-ABS-KEY(Healthy adults)) OR INDEXTERMS (“Vitamin D”) AND INDEXTERMS(BANGLADESH) AND TITLE-ABS-KEY (Prevalence) AND TITLE-ABS-KEY (Healthy adults)  (INDEXTERMS(“Vitamin D Deficiency”) AND INDEXTERMS(BANGLADESH) AND INDEXTERMS(Young adults)) OR (TITLE-ABS-KEY(“Vitamin D”) AND TITLE-ABS-KEY(BANGLADESH) AND TITLE-ABS-KEY(Young adults)) OR INDEXTERMS (“Vitamin D”) AND INDEXTERMS(BANGLADESH) AND TITLE-ABS-KEY (Prevalence) AND TITLE-ABS-KEY (Young adults)  (INDEXTERMS(“Vitamin D Deficiency”) AND INDEXTERMS(BANGLADESH) AND INDEXTERMS(adult Males)) OR (TITLE-ABS-KEY(“Vitamin D”) AND TITLE-ABS-KEY(BANGLADESH) AND TITLE-ABS-KEY(adult Males)) OR INDEXTERMS (“Vitamin D”) AND INDEXTERMS(BANGLADESH) AND TITLE-ABS-KEY (Prevalence) AND TITLE-ABS-KEY (adult Males)  (INDEXTERMS(“Vitamin D Deficiency”) AND INDEXTERMS(BANGLADESH) AND INDEXTERMS(adult Females)) OR (TITLE-ABS-KEY(“Vitamin D”) AND TITLE-ABS-KEY(BANGLADESH) AND TITLE-ABS-KEY(adult Females))  (INDEXTERMS(“Vitamin D Deficiency”) AND INDEXTERMS(BANGLADESH) AND INDEXTERMS(Premenopausal women)) OR (TITLE-ABS-KEY(“Vitamin D”) AND TITLE-ABS-KEY(BANGLADESH) AND TITLE-ABS-KEY(Premenopausal women)) | ((“Vitamin D”) OR (“Vitamin D3”)) AND ((prevalence) OR (deficiency)) AND (healthy adults) AND (Bangladesh)  ((“Vitamin D”) OR (“Vitamin D3”)) AND ((prevalence) OR (deficiency)) AND (young adults) AND (Bangladesh)  ((“Vitamin D”) OR (“Vitamin D3”)) AND ((prevalence) OR (deficiency)) AND (elderly) AND (Bangladesh)  ((“Vitamin D”) OR (“Vitamin D3”)) AND ((prevalence) OR (deficiency)) AND (adult Males) AND (Bangladesh)  ((“Vitamin D”) OR (“Vitamin D3”)) AND ((prevalence) OR (deficiency)) AND (adult Females) AND (Bangladesh)  ((“Vitamin D”) OR (“Vitamin D3”)) AND ((prevalence) OR (deficiency)) AND (Premenopausal women) AND (Bangladesh)  ((“Vitamin D”) OR (“Vitamin D3”)) AND ((prevalence) OR (deficiency)) AND (Postmenopausal women) AND (Bangladesh) |
| **PubMed** | **SCOPUS** | **Google Scholar** |
| (“Vitamin D”[MeSH]) AND Bangladesh[MeSH] AND Prevalence [MeSH] AND Premenopausal women [MeSH]  (“Vitamin D Deficiency”[MeSH]) AND Bangladesh[MeSH] AND Postmenopausal women [MeSH]  (“Vitamin D”[MeSH]) AND Bangladesh[MeSH] AND Postmenopausal women [MeSH] | INDEXTERMS(BANGLADESH) AND TITLE-ABS-KEY (Prevalence) AND TITLE-ABS-KEY (Premenopausal women)  (INDEXTERMS(“Vitamin D Deficiency”) AND INDEXTERMS(BANGLADESH) AND INDEXTERMS(Postmenopausal women)) OR (TITLE-ABS-KEY(“Vitamin D”) AND TITLE-ABS-KEY(BANGLADESH) AND TITLE-ABS-KEY(Postmenopausal women)) OR INDEXTERMS (“Vitamin D”) AND | ((“Vitamin D”) OR (“Vitamin D3”)) AND ((prevalence) OR (deficiency)) AND (Lactating women) AND (Bangladesh)  ((“Vitamin D”) OR (“Vitamin D3”)) AND ((prevalence) OR (deficiency)) AND (Elderly) AND (Bangladesh) |
| (“Vitamin D”[MeSH]) AND Bangladesh[MeSH] AND Prevalence [MeSH] AND Postmenopausal women [MeSH]  (“Vitamin D Deficiency”[MeSH]) AND Bangladesh[MeSH] AND Lactating women [MeSH]  (“Vitamin D”[MeSH]) AND Bangladesh[MeSH] AND Lactating women [MeSH]  (“Vitamin D”[MeSH]) AND Bangladesh[MeSH] AND Prevalence [MeSH] AND Lactating women [MeSH]  (“Vitamin D Deficiency”[MeSH]) AND Bangladesh[MeSH] AND Elderly[MeSH]  (“Vitamin D”[MeSH]) AND Bangladesh[MeSH] AND Elderly [MeSH]  (“Vitamin D”[MeSH]) AND Bangladesh[MeSH] AND Prevalence [MeSH] AND Elderly [MeSH]  **Same types of search terms was used for seven other south Asian countries, by replacing the country name (e.g. India, Pakistan Sri Lanka etc.) to complete search procedures.** | INDEXTERMS(BANGLADESH) AND TITLE-ABS-KEY (Prevalence) AND TITLE-ABS-KEY (Postmenopausal women)  (INDEXTERMS(“Vitamin D Deficiency”) AND INDEXTERMS(BANGLADESH) AND INDEXTERMS(lactating women)) OR (TITLE-ABS-KEY(“Vitamin D”) AND TITLE-ABS-KEY(BANGLADESH) AND TITLE-ABS-KEY(lactating women)) OR INDEXTERMS (“Vitamin D”) AND INDEXTERMS(BANGLADESH) AND TITLE-ABS-KEY (Prevalence) AND TITLE-ABS-KEY (Lactating women)  (INDEXTERMS(“Vitamin D Deficiency”) AND INDEXTERMS(BANGLADESH) AND INDEXTERMS(Elderly)) OR (TITLE-ABS-KEY(“Vitamin D”) AND TITLE-ABS-KEY(BANGLADESH) AND TITLE-ABS-KEY(Elderly)) OR INDEXTERMS (“Vitamin D”) AND INDEXTERMS(BANGLADESH) AND TITLE-ABS-KEY (Prevalence) AND TITLE-ABS-KEY (Elderly) |  |
|  |  |  |
|  |  |  |
|  |  |  |
|  |  |  |
|  |  |  |

**Table A2:** Quality assessment of selected studies (Q1, Q2, Q3……Q10 denotes ten parameters described by Hoy et al for-quality assessment)

| **Authors** | Q1 | Q2 | Q3 | Q4 | Q5 | Q6 | Q7 | Q8 | Q9 | Q10 | Total score | Quality |
| --- | --- | --- | --- | --- | --- | --- | --- | --- | --- | --- | --- | --- |
| Arya et al [27] | 1 | 1 | 1 | 0 | 0 | 0 | 0 | 0 | 1 | 0 | 4 | Moderate |
| Malhotra et al [28] | 1 | 1 | 1 | 0 | 0 | 0 | 0 | 0 | 1 | 0 | 4 | Moderate |
| Harinarayan et al [29] | 0 | 1 | 0 | 0 | 0 | 0 | 0 | 0 | 1 | 0 | 2 | Low |
| Agrawal et al [30] | 0 | 0 | 0 | 0 | 0 | 0 | 0 | 0 | 1 | 0 | 1 | Low |
| Vupputuri et al [31] | 1 | 0 | 0 | 0 | 0 | 0 | 0 | 0 | 1 | 0 | 2 | Low |
| Suryanarayana et al [32] | 1 | 0 | 0 | 0 | 0 | 0 | 0 | 0 | 1 | 0 | 2 | Low |
| Shivane et al [33] | 0 | 1 | 1 | 0 | 0 | 0 | 0 | 0 | 0 | 0 | 2 | Low |
| Harinarayan et al [34] | 1 | 1 | 1 | 0 | 0 | 0 | 0 | 0 | 1 | 0 | 4 | Moderate |
| Shukla et al [35] | 1 | 1 | 1 | 0 | 0 | 0 | 0 | 0 | 1 | 0 | 4 | Moderate |
| Sanket et al [36] | 1 | 1 | 1 | 0 | 0 | 0 | 0 | 0 | 1 | 0 | 4 | Moderate |
| Karoli et al [37] | 1 | 1 | 1 | 0 | 0 | 0 | 0 | 0 | 1 | 0 | 4 | Moderate |
| Laway et al [38] | 1 | 1 | 0 | 0 | 0 | 0 | 0 | 0 | 1 | 1 | 4 | Moderate |
| Gupta et al [39] | 1 | 1 | 0 | 0 | 0 | 0 | 0 | 0 | 1 | 0 | 3 | Low |
| Bhatt et al [40] | 0 | 0 | 0 | 0 | 0 | 1 | 0 | 0 | 1 | 1 | 3 | Low |
| Marwaha et al [41] | 1 | 1 | 1 | 0 | 0 | 0 | 0 | 0 | 1 | 0 | 4 | Moderate |
| Goswami et al [42] | 0 | 0 | 0 | 0 | 0 | 0 | 0 | 0 | 1 | 1 | 2 | Low |
| Zargar et al [43] | 1 | 1 | 1 | 0 | 0 | 0 | 0 | 1 | 0 | 0 | 4 | Moderate |
| Harinarayan et al [44] | 1 | 0 | 0 | 0 | 0 | 0 | 0 | 1 | 0 | 0 | 2 | Low |
| Sofi et al [45] | 1 | 1 | 1 | 0 | 0 | 0 | 0 | 1 | 0 | 0 | 4 | Moderate |
| Beloyartseva et al [46] | 1 | 0 | 0 | 0 | 0 | 0 | 0 | 1 | 0 | 0 | 2 | Low |
| Singh et al [47] | 0 | 1 | 1 | 0 | 0 | 0 | 0 | 1 | 0 | 0 | 3 | Low |
| Shetty et al [48] | 1 | 0 | 0 | 0 | 0 | 0 | 0 | 1 | 0 | 0 | 2 | Low |
| Kajale et al [49] | 1 | 1 | 1 | 0 | 0 | 0 | 0 | 1 | 0 | 0 | 4 | Moderate |
| Goswami et al [50] | 1 | 1 | 1 | 0 | 0 | 0 | 0 | 1 | 0 | 0 | 4 | Moderate |
| Kiran et al [51] | 1 | 1 | 1 | 0 | 0 | 0 | 0 | 1 | 0 | 0 | 4 | Moderate |
| Kumar et al [52] | 1 | 1 | 1 | 0 | 0 | 0 | 0 | 1 | 0 | 0 | 4 | Moderate |
| Goswami et al [53] | 1 | 1 | 1 | 0 | 0 | 0 | 0 | 1 | 1 | 0 | 5 | Moderate |
| Meena et al [54] | 1 | 1 | 1 | 0 | 0 | 0 | 0 | 1 | 0 | 0 | 4 | Moderate |
| Singh et al [55] | 1 | 0 | 0 | 0 | 0 | 0 | 0 | 0 | 1 | 0 | 2 | Low |
| Garg et al [56] | 1 | 0 | 0 | 0 | 0 | 0 | 0 | 0 | 1 | 0 | 2 | Low |
| Harinarayan et al [57] | 1 | 1 | 1 | 0 | 0 | 0 | 0 | 0 | 1 | 0 | 4 | Moderate |
| Paul et al [58] | 1 | 1 | 0 | 0 | 0 | 0 | 0 | 0 | 1 | 1 | 4 | Moderate |
| Srimani et al [59] | 1 | 0 | 0 | 0 | 0 | 0 | 0 | 0 | 1 | 0 | 2 | Low |
| Harinarayan et al [60] | 0 | 1 | 0 | 0 | 0 | 0 | 0 | 0 | 1 | 1 | 3 | Low |
| Agarwal et al [61] | 1 | 1 | 1 | 0 | 0 | 0 | 0 | 0 | 1 | 0 | 4 | Moderate |
| Mitra et al [62] | 1 | 1 | 1 | 0 | 0 | 0 | 0 | 0 | 1 | 0 | 4 | Moderate |
| Agarwal et al [63] | 1 | 1 | 1 | 0 | 0 | 0 | 0 | 0 | 1 | 0 | 4 | Moderate |
| Dixit et al [64] | 1 | 1 | 1 | 0 | 0 | 0 | 0 | 0 | 1 | 0 | 4 | Moderate |
| Kadam et al [65] | 1 | 1 | 1 | 0 | 0 | 0 | 0 | 0 | 1 | 1 | 5 | Moderate |
| Mahmood et al [66] | 1 | 1 | 1 | 0 | 0 | 0 | 0 | 0 | 1 | 0 | 4 | Moderate |
| Islam et al [67] | 1 | 1 | 1 | 0 | 0 | 0 | 0 | 0 | 1 | 0 | 4 | Moderate |
| Islam et al [68] | 0 | 0 | 0 | 0 | 0 | 0 | 0 | 0 | 1 | 1 | 2 | Low |
| Islam et al [69] | 1 | 0 | 0 | 0 | 0 | 1 | 0 | 0 | 1 | 1 | 4 | Moderate |
| Acherjya et al [70] | 0 | 1 | 1 | 0 | 0 | 1 | 0 | 0 | 1 | 0 | 4 | Moderate |
| **Authors** | Q1 | Q2 | Q3 | Q4 | Q5 | Q6 | Q7 | Q8 | Q9 | Q10 | Total score | Quality |
| Mubashir et al [71] | 1 | 1 | 1 | 0 | 0 | 0 | 0 | 0 | 1 | 0 | 4 | Moderate |
| Junaid et al [72] | 1 | 1 | 1 | 0 | 0 | 1 | 0 | 0 | 1 | 1 | 6 | Moderate |
| Roomi et al [73] | 1 | 1 | 1 | 0 | 0 | 0 | 0 | 0 | 1 | 0 | 4 | Moderate |
| Junaid et al [74] | 0 | 0 | 0 | 0 | 0 | 1 | 0 | 0 | 0 | 0 | 1 | Low |
| Sheikh et al [75] | 0 | 0 | 0 | 0 | 0 | 0 | 0 | 0 | 1 | 0 | 1 | Low |
| Mehboobali et al [76] | 1 | 0 | 0 | 0 | 0 | 0 | 0 | 0 | 1 | 0 | 2 | Low |
| Mansoor et al [77] | 1 | 1 | 1 | 0 | 0 | 0 | 0 | 0 | 0 | 0 | 3 | Low |
| Mustafa et al [78] | 1 | 1 | 1 | 0 | 0 | 0 | 0 | 0 | 1 | 1 | 5 | Moderate |
| Rehman et al [79] | 1 | 1 | 1 | 0 | 0 | 0 | 1 | 0 | 1 | 1 | 6 | Moderate |
| Khan et al [80] | 1 | 1 | 1 | 0 | 0 | 0 | 0 | 0 | 1 | 0 | 4 | Moderate |
| Nadeem et al [81] | 1 | 1 | 1 | 0 | 0 | 0 | 1 | 0 | 1 | 0 | 5 | Moderate |
| Iqbal et al [82] | 1 | 1 | 1 | 0 | 0 | 0 | 0 | 0 | 1 | 0 | 4 | Moderate |
| Mahmood et al [83] | 0 | 1 | 1 | 0 | 0 | 1 | 0 | 0 | 1 | 0 | 4 | Moderate |
| Afsar et al [84] | 1 | 1 | 1 | 0 | 0 | 1 | 1 | 0 | 1 | 0 | 6 | Moderate |
| Kumar et al [85] | 1 | 1 | 1 | 0 | 0 | 0 | 0 | 0 | 1 | 1 | 5 | Moderate |
| Sharif et al [86] | 1 | 1 | 1 | 0 | 0 | 0 | 0 | 0 | 1 | 1 | 5 | Moderate |
| Khan et al [87] | 1 | 1 | 1 | 0 | 0 | 0 | 0 | 0 | 1 | 1 | 5 | Moderate |
| Dar et al [88] | 1 | 1 | 1 | 0 | 0 | 0 | 0 | 0 | 1 | 0 | 4 | Moderate |
| Meyer et al [89] | 1 | 0 | 0 | 0 | 0 | 0 | 0 | 0 | 1 | 1 | 3 | Low |
| Haugen et al [90] | 1 | 0 | 0 | 0 | 0 | 1 | 0 | 0 | 1 | 1 | 4 | Moderate |
| Sherchand et al [91] | 1 | 1 | 1 | 0 | 0 | 0 | 0 | 0 | 1 | 0 | 4 | Moderate |

**
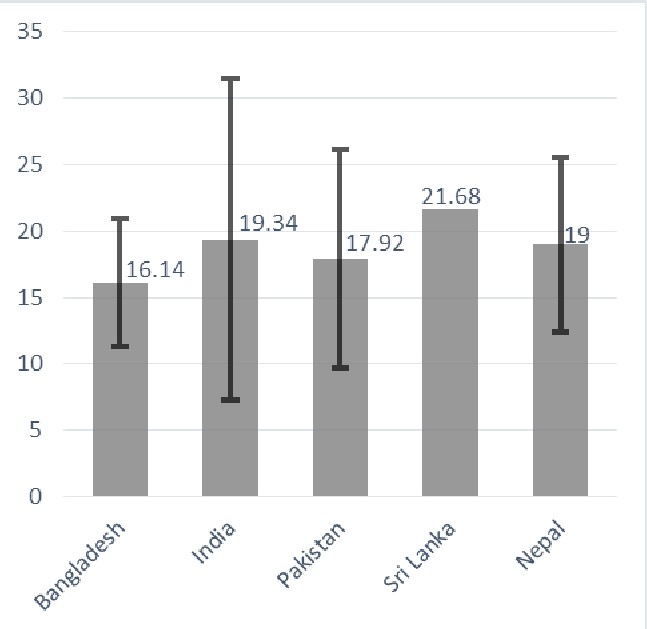
**

**Figure A1:** Weighted mean level of serum vitamin D (ng/mL) among South Asian adult population (on the Y-axis). X-axis represent the name of the South Asian countries. Error bars represent the weighted standard deviations.

**
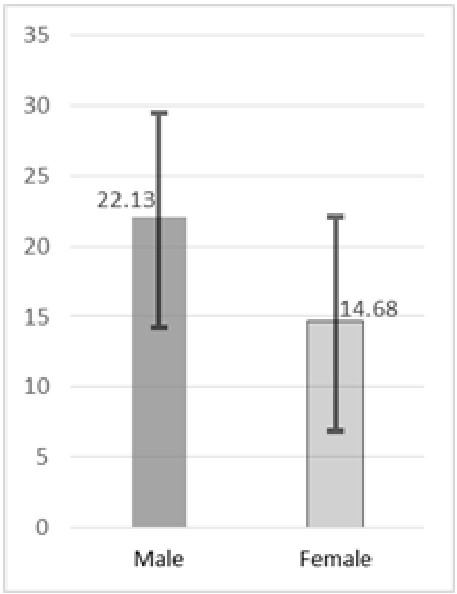
**

**Figure A2:** Weighted mean level of serum vitamin D (ng/mL; on the Y-axis) among South Asian adult male and female population. X-axis represents gender. Error bars represent the weighted standard deviations.

**Figure A3:** Funnel plot shows publication bias of the selected studies. Each dots of the funnel plot represent a single study. Y-axis represents the standard error of the effect estimates, and the X-axis shows prevalence for the corresponding studies.
